# Efficacy and safety of shenqi compound for the treatment of diabetic macroangiopathy: A protocol for systematic review and meta-analysis

**DOI:** 10.1097/MD.0000000000019682

**Published:** 2020-04-10

**Authors:** Zhipeng Hu, Maoyi Yang, Chunguang Xie, Hong Gao, Xiaoxu Fu, Hongyan Xie, Ya Liu

**Affiliations:** Hospital of Chengdu University of Traditional Chinese Medicine, Chengdu, Sichuan, P.R. China.

**Keywords:** diabetic macroangiopathy, protocol, Shen-qi compound, systematic review and meta-analysis

## Abstract

**Background:**

Diabetic macroangiopathy is a further complication of diabetes mellitus and is the leading cause of death for diabetic patients. Shenqi compound (SC) is a traditional Chinese medicine formula widely used in the treatment of diabetes and diabetic macroangiopathy. At present, there is only 1 systematic review on SC in the treatment of diabetes. However, no meta-analysis has evaluated the efficacy and safety of SC on diabetic macroangiopathy.

**Methods and analysis:**

Three English database and four Chinese medical databases will be searched from its inception to February 2020. Then 2 methodological trained researchers will screen the qualified articles by reading the title, abstract, and full texts according to an established inclusion and exclusion criteria. The assessment of risk of bias will be conducted by using the Cochrane collaboration's tool. We will conduct meta-analyses for fasting blood glucose (FBG), postprandial blood glucose (PBG), glycated hemoglobin (HbA1c), and other outcomes. The heterogeneity of data will be evaluated by Cochrane *χ* ^2^ and *I* ^*2*^ tests. We establish 3 hypotheses before the subgroup analysis actually starts: disease status at baseline, duration of intervention, type of concomitant medication. We will conduct sensitivity analysis to evaluate the stability of the results, funnel plot analysis, and Egger test to evaluate the publication bias, and assessment for the quality of evidence by the Grading of Recommendations Assessment, Development, and Evaluate system (GRADE).

**Results:**

The results will be published at a peer-reviewed journal.

**Conclusion:**

In this study, we will systematically evaluate the evidence of SC in the treatment of diabetic macroangiopathy. Our research is supposed to provide evidence-based support for clinical practice.

## 1 Introduction

Type 2 diabetes mellitus (T2DM) is a group of metabolic disorder characterized by the clinical feature of persistent hyperglycemia and pathophysiological characteristics of absolute or relative insufficiency of insulin.^[1]^ Various chronic complications may occur in the later stage of T2DM, including diabetic macroangiopathy (DM).^[2]^ DM is mainly composed of diabetic cardiovascular disease, ischemic stroke, and diabetic peripheral artery disease and is the leading cause of death for diabetic patients. According to the results of epidemiological survey, the death of more than half of diabetic patients is related to DM.^[3]^

The main cause of diabetic complications is the damage to tissues and organs caused by long-term hyperglycemia.^4^,^5^,^6^,^7^,^8^,^9^,^10^,^11^ Hyperglycemia causes macrovascular diseases through a variety of mechanisms including oxidative stress, endothelial cell injury, inflammatory response, vascular dysfunction, among others.^[12]^ At present, the main strategy for the management of DM is to decrease blood glucose so as to reduce the glucotoxicity of hyperglycemia as much as possible. However, due to the clinical inertia and the hidden incidence of diabetes, the treatment is usually delayed. Although the level of glucose can be controlled after intensive treatment, the tissue damage caused by hyperglycemia persists.^13^,^14^,^15^,^16^,^17^,^18^

In China, the application of traditional Chinese herbal medicine in DM has a long history and rich experience. SC is a traditional Chinese medicine formula which is widely used in the treatment of T2DM and DM in China. It consists of eight herbal medicine: Radix et Rhizoma Ginseng (RRG, *rén shēn*), Radix Astragali Praeparata cum Melle (RAPM, *huáng qí*), Radix et Rhizoma Salviae Miltiorrhizae (RRSM, *dān shēn*), Radix et Rhizoma Rhei (RRR, *dà huáng*), Rhizoma Dioscoreae (RD, *huái shān yào*), Radix Trichosanthis (RT, *tiān huā fĕn*), Radix Rehmanniae (RR, *dì huáng*), Fructus Corni (FC, *shān zhū yú*). Animal experiments showed that it can improve the condition of DM and the mechanisms may be related to inhibiting adiponectin, reducing inflammation among others.^19^,^20^,^21^,^22^ Furthermore, a series of clinical studies have found that it has a therapeutic effect on DM.^23^,^24^,^25^,^26^ However, these clinical studies have some shortcomings, such as small sample size, research design defects, and so on. Therefore, a high-quality systematic review and meta-analysis to summarize current clinical evidence is urgently needed.

The aim of this study is to provide an accurate presentation of the current stage of knowledge about SC in the treatment of DM and reliable evidence for clinical practice.

## 2 Methods and analysis

### 2.1 Study registration

A prospective protocol regarding the detailed search strategy and methods of data analysis was prepared according to the Preferred Reporting Items for Systematic Reviews and Meta-analysis (PRISMA) of Observation Studies in Epidemiology recommendations for study reporting. The OSF registration number is DOI 10.17605/OSF.IO/TW9MA. This systematic review and meta-analysis protocol is reported according to the Preferred Reporting Items for Systematic Reviews and Meta-analysis Protocols (PRISMA-P) checklist.^[27]^

### 2.2 Inclusion and exclusion criteria

1. Population: Adults patients with an established DM diagnose will be included in our research. There is no limitation about region, sex and age of patients.
2. Intervention: Studies that use SC as a major intervention in experimental group will be included. The control group can use any other medicines or placebo. If the authors use combination therapy of SC and other medicines in experimental group, these studies will be excluded.
3. Outcomes: Only those studies that report detailed data about DM-related outcomes will be included in our research. Studies whose outcomes are not directly related to DM will be excluded. Those studies including abstract articles, conference articles, review articles, systematic review, and meta-analysis will be excluded. For conference articles and abstract articles, we will further look for whether there are any original articles that have made further reports on their research.
4. Study design: We will only include prospective randomized controlled trials (RCTs). Observational studies like case–control studies, cohort studies and cross-sectional studies will be excluded. Similarly, retrospective studies will not be included in our research.

### 2.3 Outcomes

1. Three-point major cardiovascular adverse event (MACE) outcome (first occurrence of cardiovascular death, nonfatal myocardial infarction, or nonfatal stroke) or 5-point MACE (3-point MACE plus hospital admission for unstable angina and hospital admission for heart failure).
2. All cause death.
3. Disease-related risk score such as Framingham risk scale, China-PAR CVD Risk Calculator, risk factors for diabetes related complications.
4. FBG.
5. PBG.
6. HbA1c.
7. Total effective rate, the definition of effectiveness is elaborated in articles.
8. Ankle brachial index.
9. The peak systolic velocity of the anterior tibial artery, posterior tibial artery, and dorsalis pedis artery.
10. The inner diameter of the anterior tibial artery, posterior tibial artery, and dorsalis pedis artery.
11. The end diastolic velocity of the anterior tibial artery, posterior tibial artery, and dorsalis pedis artery.
12. The mean average velocity of the anterior tibial artery, posterior tibial artery, and dorsalis pedis artery.
13. Safety and tolerability.

### 2.4 Study search

Three English database including PubMed, Embase, Cochrane Library Central Register of Controlled Trials, and 4 Chinese databases including China National Knowledge Infrastructure (CNKI) database, Wanfang Data Knowledge Service Platform, the VIP information resource integration service platform (cqvip), China Biology Medicine Disc (Sino Med) will be searched from their inception to January 2020 with a language limitation of English and Chinese. In addition, Google scholar and Baidu Scholar will be used to find out potential missing papers. There is no time limitation about literatures. The Chinese Clinical Trial Registry (ChiCTR) and ClinicalTrials.gov will be searched to ensure that no clinical studies are missed.

To obtain literature as comprehensively as possible, we will retrieve all the literature related to SC and then screen it according to inclusion and exclusion criteria. The search terms used will be as follows: “shenqi fufang,” “shenqi compound,” “SHEN-QI compound,” “shenqi formula,” “shenqi compound formula,” “Shen-Qi Compound formula,” “Shen-Qi formula.” Two authors (ZH and MY) will search and screen all the citations independently. An example of search process is presented in Table 1.

**Table 1:**
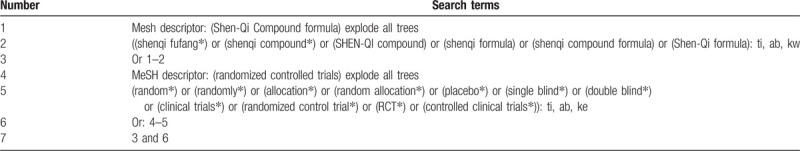
Example of Cochrane search strategy.

### 2.5 Study selection

**Figure 1:**
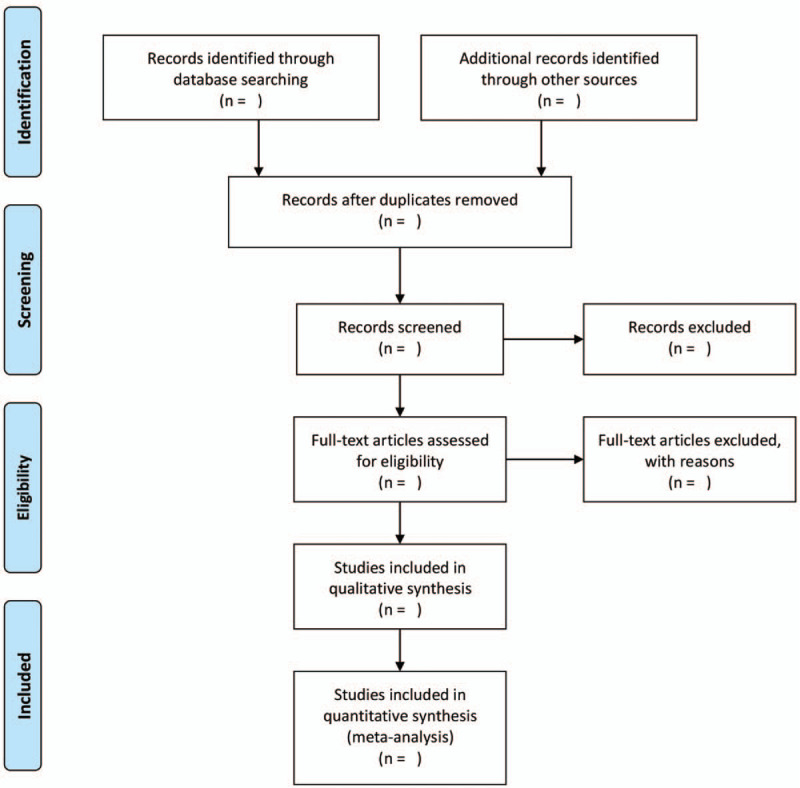
Flow chart of study selection.

We will import the documents we download from the database into Endnote X8 for Mac (Thomson Reuters) software and then 2 methodological trained researchers will screen the qualified articles by reading the title and abstract according to the inclusion and exclusion criteria. Those documents that meet the criteria will be downloaded for further reading. A final decision will be made through consensus when there are discrepancies between researchers. A flow chart will be drawn to show the process of study selection (Fig. 1).

### 2.6 Data extraction

The data of those qualified articles will be extracted into Microsoft Excel according to a predefined form for further analysis. For those studies with incomplete data, we will contact the authors for more detailed information. If we cannot contact the authors or the authors refuse to provide detailed information, then the document will be excluded. For those literature with the same data as another articles, for instance, repeatedly published articles, we will choose the one with more detailed data.

For each study, the following information will be extracted: title, the publication country, the first authors of the article, time of publication, baseline information of participants, interventions in experimental group, interventions in control group, time and dose of treatment, course of disease, number of patients in each group, ages and sex of patients, outcomes, safety, and tolerability.

### 2.7 Risk of bias assessment

The risk of bias of included studies will be assessed by using the Cochrane collaboration's tool. In this tool, the risk of bias of a study is assessed from 7 aspects: random sequence generation (selection bias), allocation concealment (selection bias), blinding of participants and personnel (performance bias), blinding of outcome assessment (detection bias), incomplete outcome data (attrition bias), selective reporting (reporting bias), other bias.^[28]^ Each item is classified as “Low risk,” “High risk,” or “Unclear risk.” If the experimental process is not described in detail in the article, we will contact the authors by E-mail for more methodological information. Two reviewers will conduct the risk of bias assessment independently and any disagreements will be solved by consensus.

### 2.8 Data analysis

Data analysis will be conducted in Review Manager Version 5.3 and stata 14.0 software for Mac. The former is mainly used to create forest plots and conduct subgroup analysis and the latter is used to conducted sensitivity analysis and publication bias. For the binary variable, the effect size will be represented as risk ratio (RR) and 95% confidence interval (CI) and a Der-Simonian-Laird method will be used to calculate them. For continuous variable, the effect size will be represented as mean difference (MD) and 95% CI. The heterogeneity of data will be investigated by Cochrane *χ* ^2^ and *I* ^*2*^ tests. The statistical heterogeneity is considered substantial when *P* < .05 and *I* ^*2*^ > 50% and the random-effect model will be applied to pool data. If there is no significant heterogeneity (*P* > .05 and *I* ^*2*^ < 50%), then the fixed-effect model will be used to calculate the effect size. If quantitative synthesis is not appropriate due to substantial heterogeneity, then systematic review will be conducted and the results will be presented with tables and figures.

### 2.9 Investigation of heterogeneity

If there is substantial heterogeneity between studies, then we will conduct subgroup analysis to explore the heterogeneity. To avoid post hoc analysis, the subgroup analysis will be conducted according to 3 hypotheses: baseline condition, duration of intervention, type of concomitant medication. To further improve the reliability of subgroup analysis, we will evaluate the credibility of our subgroup analysis according to the guidance for credible subgroup analysis. If there are enough studies included, then meta-regression will be conducted to further explore the heterogeneity. Those subgroup effects that occur simultaneously in subgroup analysis and regression analysis will be considered credible.

### 2.10 Sensitivity analysis

To ensure the stability of the results, we will conduct sensitivity analysis of the results by excluding each of the studies included in the analysis one by one, then re-analyzing the results, and comparing the differences between the re-obtained results and the original results. In this way, we will be able to assess the impact of individual studies on overall outcomes and their robustness.

### 2.11 Reporting bias assessment

The integrity of the studies is an important factor affecting the accuracy of the results and conclusions of meta-analysis. The integrity of the included studies is mainly measured by reporting bias, of which publication bias is the most common. Therefore, this study will identify report bias by publication bias assessment. A funnel plot will be drawn to investigate the publication bias. Funnel plot will be asymmetric when publication bias exists, such as when research with small sample and no statistically significant results are not published. The more obvious the asymmetry of funnel plot is, the more likely there is publication bias.^[29]^ And then Egger test will be conducted for statistical assessment the publication bias. The publication bias is considered to exist if *P* < .05.^[30]^

### 2.12 Summary of finding tables

Finally, this study will evaluate the quality of evidence for each outcome through Grading of Recommendations Assessment, Development and Evaluate system (GRADE), the most widely used evidence quality classification standard at present.^[31]^ RCTs will be defined as high-level evidence and observational studies as low-level evidence in GRADE system. Researchers can downgrade the quality of evidence in RCTs from a high level to a moderate or lower level depending on whether there are factors affecting the quality of evidence.

The factors affecting the quality of evidence include the risk of bias in a single study (methodological quality), the indirectness of evidence, the heterogeneity of research results, the accuracy of effect estimation, and publication bias.

### 2.13 Patient and public involvement

There is no patient and public involved in this study.

### 2.14 Ethics and dissemination

Ethical approval is not needed for this meta-analysis. This study comprehensively evaluates the existing research evidence of SC and can provide evidence-based medical support for clinical workers. The results of our research will be published in a peer-reviewed journal.

## 3 Discussion

DM is the leading cause of death in T2DM patients. The purpose of this study is to summarize the clinical evidence of SC in the treatment of DM.

To avoid bias as much as possible, we collect all relevant documents as comprehensively as possible. As to the exploration of heterogeneity, post hoc subgroup analysis should be avoided as much as possible. Therefore, this study presupposes several subgroup hypotheses before the study actually starts. In addition, we will evaluate the reliability of subgroup analysis, which is lacking in most studies at present. Finally, we will use GRADE system to assess the evidence so as to contribute to a better guide for clinical practice.

### 3.1 Amendments

If any modification is required, we will update our protocol to include any changes in the entire research process.

## Author contributions

**Conceptualization:** Zhipeng Hu, Maoyi Yang, Chunguang Xie.

**Data curation:** Hong Gao, Xiaoxu Fu.

**Formal analysis:** Zhipeng Hu, Maoyi Yang.

**Funding acquisition:** Chunguang Xie.

**Investigation:** Hongyan Xie.

**Methodology:** Zhipeng Hu, Maoyi Yang, Chunguang Xie.

**Project administration:** Chunguang Xie.

**Resources:** Zhipeng Hu, Chunguang Xie.

**Software:** Zhipeng Hu, Maoyi Yang.

**Supervision:** Hongyan Xie.

**Writing – original draft:** Zhipeng Hu.

**Writing – review & editing:** Chunguang Xie, Ya Liu.

Zhipeng Hu orcid: 0000-0003-1524-6452.
